# Benzodiazepines induce hyperglycemia in rats by affecting peripheral disposal of glucose

**DOI:** 10.1186/cc12323

**Published:** 2013-03-19

**Authors:** VS Salice, FV Valenza, MP Pizzocri, LV Valenti, GC Chevallard, MU Umbrello, SG Gatti, SF Fargion, GI Iapichno, LG Gattinoni

**Affiliations:** 1 Università degli Studi Milano, Milan, Italy

## Introduction

In light of the interest in the relationship between glycemia control in critically ill subjects and outcome, we set up a study to investigate whether benzodiazepine, commonly used in anesthesia and ICUs, interferes with glucose metabolism and to explore the mechanism.

## Methods

A total of 40 sedated and paralyzed Sprague-Dawley rats (301 ± 55 g) were investigated in four consecutive studies. (1) To investigate the effects of diazepam on blood glucose, 15 rats were randomly assigned to intraperitoneal anesthesia with tiopenthal 80 mg/kg (DZP0), tiopenthal 40 mg/kg + diazepam 5 mg/kg (DPZ5) or tiopenthal 40 mg/kg + diazepam 15 mg/kg (DZP15). Blood levels of glucose (GEM premier 3000; IL) were measured at time intervals over 2 hours. (2) Ten animals randomized to DZP0 or DZP5 underwent an intravenous glucose tolerance test with glucose bolus (0.5 g/kg). Acute insulin response, the mean value of blood insulin (Insulin ELISA kit; Millipore) from 2 to 10 minutes after glucose bolus, was measured as index of insulin secretion. (3) A hyperinsulinemic euglycemic clamp obtained by a continuous intravenous infusion of insulin (130 mUI/kg/minute) was run in 10 animals randomized to DZP0 or DZP5 and the glucose infusion rate (GIR, mg/kg/minute) was assessed [1]. (4) The effect of midazolam on blood glucose was tested in five additional animals (M5: tiophental 40 mg/kg + midazolam 5 mg/kg). Data are presented as mean ± SEM. Statistical analysis (ANOVA, *t *test) was conducted with Sigma Stat 3.1 (Systat Software).

## Results

(1) Diazepam was associated with higher levels of blood glucose in a dose-response fashion: DZP0 128 ± 7 mg/dl, DZP5 166 ± 7.3, DZP15 197 ± 7 (*P <*0.05). (2) The acute insulin response to intravenous glucose tolerance test was similar in DZP0 and DZP5 (DZP0 3.97 ± 0.42 ng/ml, DZP5 3.68 ± 0.44, *P *= 0.68), while blood glucose levels were different (DZP0 192 ± 5 mg/dl, DZP5 217 ± 5, *P <*0.05). (3) During hyperinsulinemic euglycemic clamp, blood glucose levels were similar (109 ± 3 mg/dl, *P *= 0.2), but the DZP5 group showed a trend through lower values of GIR (DZP0 30.8 ± 2 mg/kg/minute, DZP5 24.7 ± 2, *P *= 0.08). (4) Infusion of midazolam was associated with higher blood glucose levels (DZP0 128 ± 5 mg/dl, M5 151 ± 6, *P <*0.05).

## Conclusion

Both diazepam and midazolam significantly alter plasma glucose levels in rats. Peripheral disposal of glucose rather than altered pancreas secretion of insulin explains the benzodiazepine-associated hyperglycemia.
